# Elevated HDAC4 Expression Is Associated with Reduced T-Cell Inflamed Tumor Microenvironment Gene Signatures and Immune Checkpoint Inhibitor Effectiveness in Melanoma †

**DOI:** 10.3390/cancers17091518

**Published:** 2025-04-30

**Authors:** Mariam K. Alamoudi, Abdulmonem A. Alsaleh, Anita Thyagarajan, Faisal K. Alkholifi, Muhammad Liaquat Raza, Ravi P. Sahu

**Affiliations:** 1 Department of Pharmacology, College of Pharmacy, Prince Sattam Bin Abdulaziz University, Al-Kharj 11942, Saudi Arabia; f.alkholifi@psau.edu.sa; 2 Blood and Cancer Research Department, King Abdullah International Medical Research Center (KAIMRC), King Saud bin Abdulaziz University for Health Sciences (KSAU-HS), Ministry of National Guard-Health Affairs (MNG-HA), Riyadh 11481, Saudi Arabia; alsalehabd@kaimrc.edu.sa; 3 Department of Pharmacology and Toxicology, Boonshoft School of Medicine, Wright State University, Dayton, OH 45435, USA; anita.thyagarajan@wright.edu; 4 Department of Infection Prevention Control, King Abdullah International Medical Research Center (KAIMRC), King Saud bin Abdulaziz University for Health Sciences (KSAU-HS), Ministry of National Guard Health Affairs (MNG-HA), Riyadh 11481, Saudi Arabia; razamu2@mngha.med.sa

**Keywords:** melanoma, immune checkpoint inhibitor, HDAC inhibitor, tumor microenvironment

## Abstract

**Background/Objectives**: Melanoma remains a difficult malignancy to treat because it employs tolerance mechanisms like negative immune checkpoint (IC) molecules to avoid antitumor immune responses. Thus, immune checkpoint inhibitors (ICIs) are increasingly used to treat melanoma. However, many patients do not respond, indicating resistance mechanisms like intrinsic tumor characteristics and an immunosuppressive tumor microenvironment (TME). An inflamed TME was associated with improved ICI efficacy by upregulating the T-cell inflamed TME gene signatures, an array of genes associated with dendritic cells (DCs) and cytotoxic CD8^+^ T-cell-mediated anti-tumor responses. As histone deacetylases (HDACs) have been shown to play crucial roles in regulating gene expression and aberrant HDAC expression has been reported in melanoma and also implicated in the regulation of IC, programmed cell death protein 1 (PD-1), and its ligand (PD-L1) and various immune evasion genes, we investigated the relationship between T-cell inflamed TME gene signatures and the HDAC family, particularly HDAC4. **Methods**: We used the skin cutaneous melanoma (SKCM) database, ICI-pretreated melanoma dataset, and other platforms including cBioPortal, TIMER 2.0, TISIDB, and UALCAN for the analysis. **Results**: We identified that high HDAC4 expression negatively modulated the TME by decreasing the abundance of DCs and cytotoxic CD8^+^ T-cells. The group of melanoma patients with elevated HDAC4 expression exhibited not only poor prognosis but also diminished transcription of T-cell inflamed TME gene signatures and increased DNA methylation of T-cell inflamed TME gene signatures. Importantly, elevated HDAC4 expression was associated with decreased CD8^+^ T-cells and a decreased ESTIMATE immune score in ICI-pretreated melanoma patients. **Conclusions**: Our findings suggest that HDAC4 may transform the TME into a non-inflamed phenotype, thereby reducing ICI efficacy in melanoma. Overall, this research shows that a combination of HDAC4 inhibitors and ICIs could result in better melanoma prognosis.

## Simple Summary

While multiple therapeutic options exist for melanoma, their overall response rates in the majority of patients remain relatively low due to the interference of counter-regulatory mechanisms. This indicates the need to understand the underlying mechanisms and devise alternative approaches to enhance the effectiveness of melanoma therapies. Given the importance of genes that regulate tumor immune responses, the aim of our study was to determine the role of one such gene named histone deacetylase 4 (HDAC4) with the objective of evaluating its impact in the tumor microenvironment (TME). Using genomic databases, we found that increased levels of HDAC4 were associated with decreased antitumor immune responses, resulting in the reduced overall survival and disease-free survival of melanoma patients. Overall, these findings suggest that HDAC4 inhibitors could be explored to improve the antitumor immune responses and/or effectiveness of immunotherapy in melanoma patients.

## 1. Introduction

Over the last few decades, the incidence of melanoma has been increasing worldwide [1,2]. Compared to other skin cancers such as squamous and basal cell carcinomas, melanoma is highly aggressive in nature and often diagnosed with advanced-stage or metastatic disease and has a poor prognosis [1,2,3,4,5]. Among common etiological factors, exposure to ultraviolet (UV) radiation, family history, and genetic alterations have been linked with an increased risk of developing melanoma [1,2,6,7,8,9]. Notably, despite multiple ongoing therapeutic options, their response rates in the majority of melanoma patients remain relatively low due to the interference of counter-regulatory mechanisms, including the development of tumor resistance mechanisms [10,11,12,13,14,15,16,17,18,19,20,21].

Since the development of tumor resistance also depends on host antitumor responses that employ immunosurveillance mechanisms to prevent and facilitate the regression of established tumors, the characteristics of immune features (hot, variable, and cold) and tumor microenvironments (TMEs) play critical roles in defining the efficacy of therapeutic agents [22,23,24,25]. In addition, immune cell types such as macrophages, natural killer (NK) cells, myeloid-derived suppressor cells (MDSCs), B-lymphocytes, and T-lymphocytes, including T-helper-1 (TH_1_) and 2 (TH_2_), regulatory T-cells (Tregs), and cytotoxic T-cells (CTLs), all constitute the TME [26,27]. Importantly, melanoma is an immunologically hot tumor, which is characterized by a TME rich in tumor-infiltrating lymphocytes (TILs such as cytotoxic CD8^+^ T-cells and NK cells), immune checkpoint (IC) ligands such as programmed death ligand 1 (PD-L1) overexpression on tumor cells, genomic instability, and pre-existing antitumor immune response [28,29]. These hallmarks of hot tumors predict treatment efficacy. Moreover, other factors, including tertiary lymphoid tissues (TLTs) characterized by ectopic lymphoid tissues that drive antigen-specific immune responses at sites of chronic inflammation, microsatellite status, tumor mutational burden (TMB), ICs, T-cell immunoglobulin and mucin-domain containing-3 [TIM-3], and lymphocyte activation gene-3 [LAG3], the inhibitory receptor expressed on T-cells or other immune cells, all influence treatment efficacy [28,29,30].

As melanoma tumors evade antitumor immune responses by employing tolerance mechanisms such as IC molecules, immune checkpoint inhibitors (ICIs), including anti-PD-1 (nivolumab and pembrolizumab) and anti-PD-L1 (atezolizumab), have been widely used as a single agent or in combination with BRAF/MEK inhibitors to treat melanoma patients [31,32,33]. While a reduction in tumor growth and metastasis as well as an overall increase in survival benefits are documented, these ICIs are associated with a lower response rate in melanoma patients [34,35]. To that end, T-cell inflamed TME gene signatures, which are characterized by the infiltration of antigen-specific T-cells, type I and type II interferon (IFN)-associated gene transcripts, dendritic cells (DCs), and the promigratory chemokine-dependent recruitment of CD8^+^ effector T-cells, have been reported to be the most robust predictor of immunotherapy efficacy [36,37,38,39,40,41,42]. Importantly, different T-cell inflamed TME gene signatures, including the T-cell inflamed signature, type II IFNγ-related gene signature, and T effector signature, have been identified [38,39,40,41,42]. They are associated with a favorable clinical response to ICIs among multiple tumor types, including melanoma [38,39,40,41,42]. Therefore, these T-cell inflamed TME gene signatures may serve as a predictive biomarker for assessing patient samples and guiding the application of ICIs.

Given that histone deacetylases (HDACs) play crucial roles in regulating gene expression and aberrant HDAC expression is frequently documented in melanomas, it has been demonstrated that HDACs can modulate the transcription of PD-1/PD-L1 and other associated genes linked to immune evasion [43,44,45,46,47,48]. Of various HDAC isoforms, HDAC1, 2, 4, and 6 are linked with clinicopathological parameters such as increased tumor size and a higher mitotic index in patients with uveal melanomas [49]. To ascertain which HDAC isoforms play a role in SKCM, we investigated prognostic markers within the SKCM dataset in UALCAN and found that the group of patients with low HDAC4 expression has a favorable outcome. However, the association between HDAC4 and T-cell inflamed TME gene signatures as well as how they influence ICI efficacy have not been described. In addition, while the effect of HDAC4 DNA methylation or HDAC4 expression on enhancer DNA methylation has been correlated with other pathological conditions [50,51], the impact of HDAC4 expression on the DNA methylation of T-cell inflamed TME gene signatures has not been studied. To that end, the objective of our research was to examine the association between the T-cell inflamed TME gene signatures and HDACs, with particular focus on HDAC4 as well as how HDAC4 expression influences DNA methylation and the transcription of T-cell inflamed TME gene signatures in melanoma patients. We also utilized an ICI-pretreated melanoma cohort to further investigate the role of HDAC4.

## 2. Materials and Methods

### 2.1. The Co-Expression Study Between the HDAC4 Gene and the T-Cell Inflamed TME Signature Genes

The study was conducted using the Skin Cutaneous Melanoma (SKCM) (TCGA, Firehose Legacy) dataset in cBioPortal (https://www.cbioportal.org/; accessed on 11 November 2024) [52,53,54]. The HDAC4 gene and the T-cell inflamed TME gene signatures we analyzed include the following:

1. T-cell inflamed signature: interferon regulatory factor 1 (IRF1), CD8A, granzyme K (GZMK), inducible T-cell co-stimulator (ICOS), C-C motif chemokine ligands (CCL3, CCL4), C-X-C motif chemokine ligands (CXCL9, CXCL10), and major histocompatibility complex, class II (HLA-DMA, HLA-DMB, HLA-DOA, HLA-DOB) [38,39].
2. Type II IFN-γ-related gene signature: CD8A, PD-L1 (also known as CD274), chemerin chemokine-like receptor 1 (CMKLR1), CCL5, CXCL9, C-X-C motif chemokine receptor 6 (CXCR6), HLA-DQA1, HLA-DRB1, natural killer cell granule protein 7 (NKG7), proteasome 20S subunit, beta type, 10 (PSMB10), lymphocyte activation gene 3 (LAG3), indoleamine 2,3-dioxygenase 1 (IDO1), T-cell immunoreceptor with Ig and ITIM domains (TIGIT), and signal transducer and activator of transcription 1 (STAT1) [38,40].
3. T effector signature: CD8A, CD4, GZMA, GZMB, perforin 1 (PRF1), IFNG, ICOS, CXCL9, and CXCL10 [38,41,42].

These gene signatures were queried (or entered into the dataset), and their mRNA expression levels were examined. The Co-expression tab in cBioPortal was utilized to investigate the correlation between the mRNA expression (RNA Seq V2 RSEM) of HDAC4 and T-cell inflamed TME gene signatures in the patient dataset (n = 287).

To study the co-expression between HDAC4 and T-cell inflamed TME gene signatures using a heat map and hierarchical clustering, mRNA expression z-scores relative to all samples (log RNA Seq V2 RSEM) of HDAC4 and T-cell inflamed TME gene signatures were retrieved from the SKCM (TCGA, Firehose Legacy) dataset in cBioPortal (n = 472) (https://www.cbioportal.org/; accessed on 11 November 2024) [52,53,54]. The data were generated using the Morpheus online platform (https://software.broadinstitute.org/morpheus/; accessed on 13 November 2024).

Further investigation was conducted to examine the correlation between HDAC4 ex-pression and the expression of T-cell inflamed TME gene signatures. The TISIDB database (http://cis.hku.hk/TISIDB/; accessed on 9 April 2025), which contains the SKCM from the TCGA dataset, was employed [55]. The data were extracted from the immunomodulator and chemokine sections to assess Spearman’s correlations between HDAC4 expression and T-cell inflamed TME gene signatures that include the T-cell inflamed signature: ICOS, CCL3, CCL4, CXCL9, CXCL10, HLA-DMA, HLA-DMB, HLA-DOA, and HLA-DOB [38,39]; the Type II IFN-γ-related gene signature: PD-L1 (CD274), CCL5, CXCL9, CXCR6, HLA-DQA1, HLA-DRB1, LAG3, and IDO1 [38,40]; and the T effector signature: ICOS, CXCL9, and CXCL10 [38,41,42]. The data for the remaining genes of the T-cell inflamed TME gene signatures were unavailable in TISIDB.

### 2.2. Immune Cell Association Study

The SKCM from the TCGA dataset (n = 471), acquired from TIMER 2.0 (http://timer.cistrome.org/; accessed on 3 December 2024), was utilized for the assessment [56,57,58]. The correlation between HDAC4 expression and the infiltration of myeloid DC (mDC), plasmacytoid DC (pDC), and cytotoxic CD8^+^ T-cells, along with the association between T-cell inflamed TME gene signatures and these immune cells, was investigated utilizing multiple algorithms (XCELL, MCP-COUNTER, TIMER, CIBERSORT, CIBERSORT-ABS, EPIC, and QUANTISEQ).

The TISIDB database (http://cis.hku.hk/TISIDB/; accessed on 3 December 2024), which contains the SKCM from the TCGA dataset, was utilized to examine the association between HDAC4 expression and the expression of T-cell inflamed TME gene signatures in relation to the abundance of TILs, including activated DCs and cytotoxic CD8^+^ T-cells [55].

### 2.3. The Transcription Analysis and the DNA Methylation Analysis of T-Cell Inflamed TME Signature Genes with Regard to HDAC4 Expression

To investigate the effect of HDAC4 expression on the mRNA expression and the DNA methylation levels of the T-cell inflamed TME gene signatures, we used the SKCM (TCGA, Firehose Legacy) dataset in cBioPortal (https://www.cbioportal.org/; accessed on 11 November 2024) [52,53,54]. Patients were divided into two groups based on HDAC4 mRNA expression relative to all samples using a z-score threshold of 1: high HDAC4 expression (HDAC4: EXP > 1, n = 64) and low HDAC4 expression (HDAC4: EXP < −1, n = 71). The data for the transcription analysis and DNA methylation analysis were downloaded from the mRNA tab and the DNA methylation tab in the Comparison/Survival module of cBioPortal, respectively.

### 2.4. Survival Prognosis Analysis

We used the SKCM (TCGA, Firehose Legacy) dataset in cBioPortal (https://www.cbioportal.org/; accessed on 11 November 2024) [52,53,54]. For survival prognosis analysis, we utilized HDAC4 mRNA expression relative to all samples, and a z-score threshold of 1 was applied to classify the patients. The overall survival (OS) and disease-free survival (DFS) data were downloaded using the Survival tab in the Comparison/Survival module of cBioPortal. Moreover, to enhance the generalizability of our investigation, the data of patients exhibiting HDAC4 mRNA expression z-scores relative to all samples between −1 and 1 were obtained.

We performed further investigation using the UALCAN database (https://ualcan.path.uab.edu/index.html; accessed on 11 November 2024) to assess the effect of HDAC4 and T-cell inflamed TME gene signatures on the survival of SKCM patients [59,60]. The median of gene expression was used to classify the patients. Kaplan–Meier curves with log-rank *p*-values were downloaded from the SKM dataset (TCGA) on the UALCAN website.

### 2.5. The Effect of HDAC4 Expression in ICI-Pretreated Melanoma Patients

We used the melanoma (MSK, NEJM 2014) dataset from the immunogenomic studies section of cBioPortal (https://www.cbioportal.org/; accessed on 10 April 2025) [52,53,54]. The dataset contains whole-exome sequencing of pretreated (ipilimumab or tremelimumab) melanoma tumor–normal pairs [61]. We categorized the patients according to their CBSET T-cell CD8 levels into low (n = 10) and high (n = 11) CBSET T-cell CD8 groups. Moreover, we classified the patients according to the ESTIMATE immune score into two groups: low (n = 10) and high (n = 11) ESTIMATE immune score groups. To investigate the impact of HDAC4 expression on TIL status, HDAC4 mRNA expression data were obtained for the high and low CBSET T-cell CD8 groups as well as for the high and low ESTIMATE immune score groups from the mRNA tab in the Comparison/Survival module of cBioPortal.

### 2.6. Statistical Analysis

Spearman’s correlation coefficient was used to assess the co-expression study and immune cell association study. Student’s *t*-test was used for the comparison between two groups in the transcription analysis, the DNA methylation analysis, the CBSET T-cell CD8 analysis, and the ESTIMATE immune score analysis. Survival analysis, both OS and DFS, was performed utilizing the log-rank test. Kaplan–Meier plots were generated utilizing GraphPad Prism 10.0. Statistical significance was defined as a *p*-value less than 0.05.

## 3. Results

### 3.1. The Co-Expression Profile of HDAC4 and T-Cell Inflamed TME Gene Signatures

**Figure 1:**
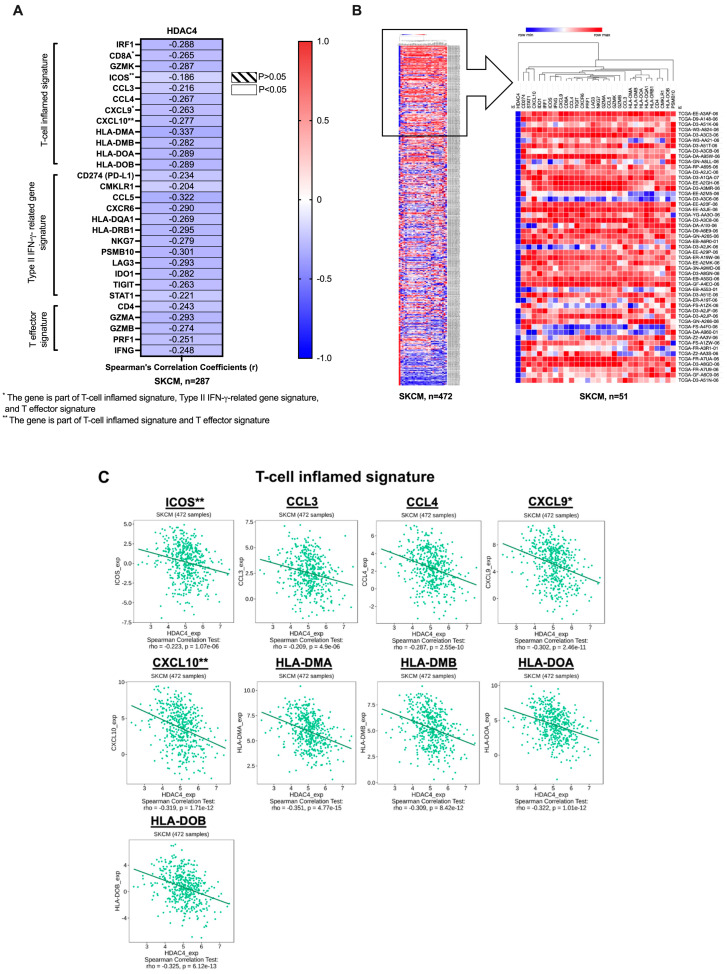

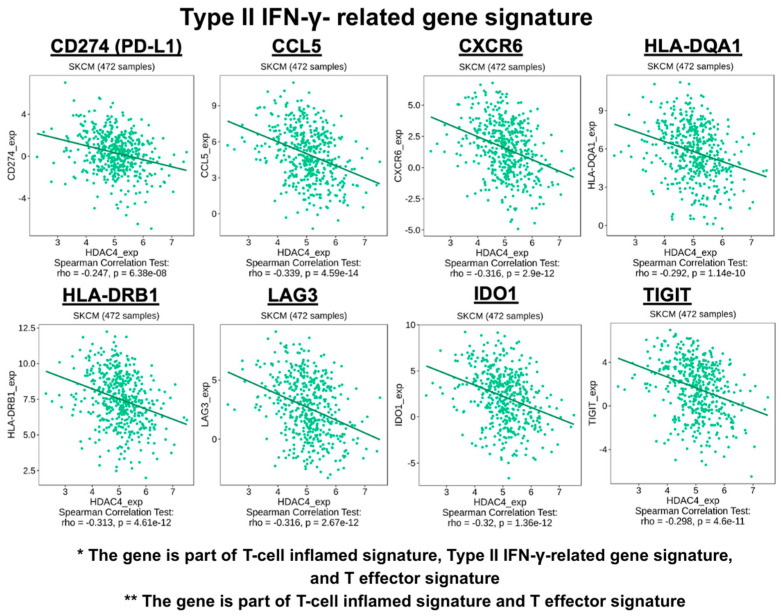
HDAC4 negatively co-expressed with T-cell inflamed TME gene signatures. (**A**) The correlation between mRNA expression of HDAC4 and T-cell inflamed TME gene signatures. Each cell represents the spearman’s coefficient r-value, which indicates the patient samples. The blue cell indicates a negative correlation. (**B**) The heat map shows the mRNA expression z-scores relative to all samples for HDAC4 and T-cell inflamed TME gene signatures in columns against each patient sample in rows. A cluster of melanoma patients (n = 51) with low expression of HDAC4 and high expression of T-cell inflamed TME gene signatures was indicated. (**C**) Spearman’s coefficient rho value, which indicates the patient population, for Spearman’s correlation between mRNA expression of HDAC4 and T-cell inflamed TME gene signatures are identified. A *p*-value < 0.05 is considered statistically significant.

We first determined the mRNA co-expression profile between HDAC4 and T-cell inflamed TME gene signatures, including the T-cell inflamed signature, type II IFNγ related gene signature, and T effector signature, as detailed in the Materials and Methods section [38,39,40,41,42]. The analysis was conducted utilizing the SKCM (TCGA, Firehose Legacy) dataset in cBioPortal (n = 287). As shown in Figure 1A, there was a negative correlation between HDAC4 and all the T-cell inflamed TME gene signatures analyzed, suggesting an inverse relationship. Overall, these findings suggested that as HDAC4 expression increased, the expression of the T-cell inflamed TME gene signatures decreased.

We further confirmed our results utilizing heat map and hierarchical clustering of gene expression data. The resulting clustering divided the data into two distinct groups: one arm containing HDAC4 and the other arm containing the T-cell inflamed TME gene signatures we investigated (Figure 1B and Figure S1). This division underscored the negative correlation between HDAC4 and T-cell inflamed TME gene signatures. A distinct cluster of patients was identified (n = 51), characterized by low HDAC4 expression and high T-cell inflamed TME gene signature expression, supporting the inverse relationship between these gene sets (Figure 1B). Figure S1 provides the full dataset used for the correlation and clustering analyses (n = 472), highlighting the negative correlation between HDAC4 and T-cell inflamed TME gene signatures across the sample population.

Additional correlation analysis was conducted utilizing the SKCM dataset within the TISIDB database. Figure 1C indicated an opposite relationship between HDAC4 expression and the analyzed T-cell inflamed TME gene signatures, as specified in the Materials and Methods section.

### 3.2. The Effect of HDAC4 Expression and T-Cell Inflamed TME Genes Signature Expression on the TME

**Figure 2:**
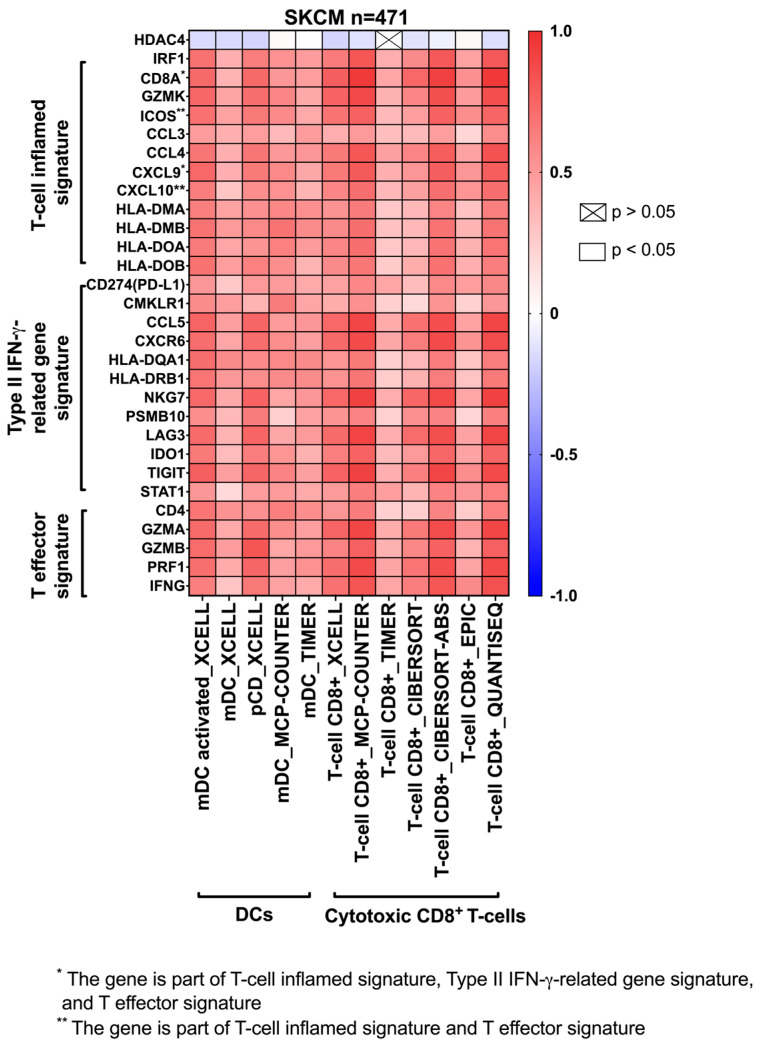
HDAC4 negatively correlated with the infiltration of mDCs, pDCs and cytotoxic CD8^+^ T-cells into the TME, while T-cell inflamed TME gene signatures positively correlated with the infiltration of mDCs, pDCs and cytotoxic CD8^+^ T-cells into the TME. Each cell represents the Spearman’s coefficient rho value, which indicates the patient population. A red cell represents a positive correlation, while a blue cell indicates a negative correlation. A *p*-value < 0.05 is considered statistically significant.

Employing the SKCM dataset obtained from TIMER 2.0, this study aimed to explore the relationship between HDAC4 expression, T-cell inflamed TME gene signature expression, and the infiltration of mDCs, pDCs, and cytotoxic CD8^+^ T-cells (Figure 2). The results demonstrated a negative correlation between HDAC4 expression and the infiltration of both mDCs and cytotoxic CD8^+^ T-cells, indicating that higher HDAC4 expression was associated with reduced immune cell infiltration. Furthermore, the data showed that there was a positive association between the T-cell inflamed TME gene signature expression we examined and the infiltration of both mDCs as well as cytotoxic CD8^+^ T-cells, suggesting their significance in the development of an inflamed TME. Furthermore, the data showed that the infiltration of pDCs, which are a distinct population of DCs that secret both type I and type II IFN, was negatively influenced by HDAC4 expression. However, pDC infiltration exhibited a positive correlation with the expression of genes associated with T-cell inflamed TME signatures. Overall, these findings suggested that overexpression of HDAC4 could negatively impact tumor immunogenicity.

**Figure 3:**
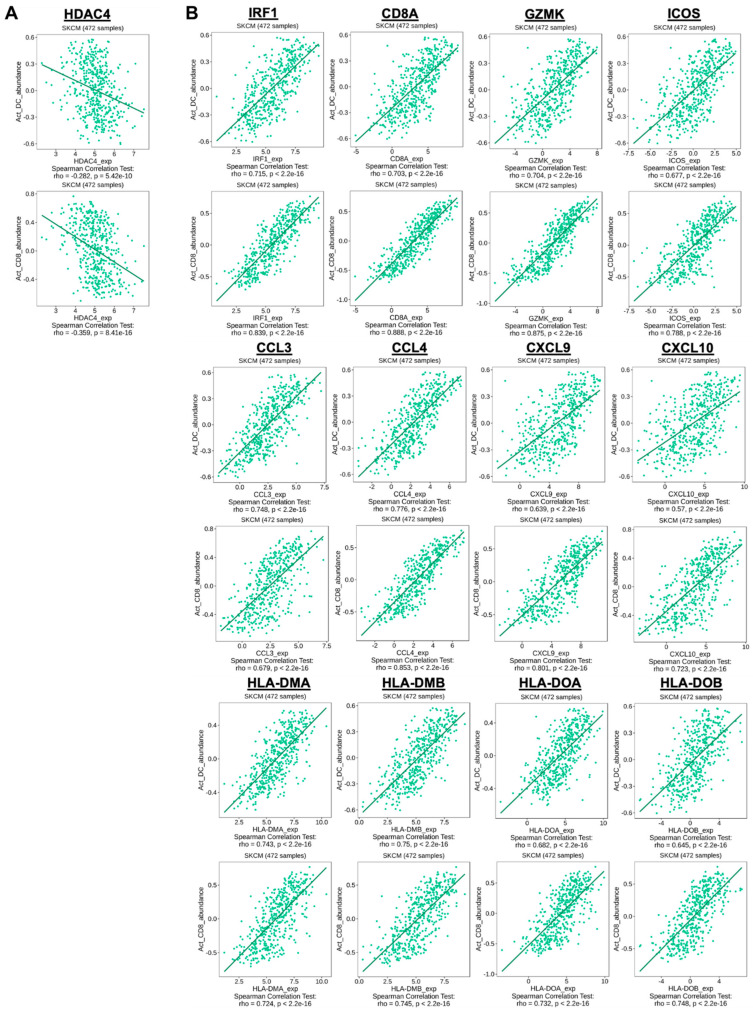
High HDAC4 expression exhibited an adverse association with the abundance of activated DCs and activated cytotoxic CD8^+^ T-cells within the TME, whereas high T-cell inflamed signature expression demonstrated a favorable association with the abundance of activated DCs and activated cytotoxic CD8^+^ T-cells within the TME. (**A**,**B**) Spearman’s coefficient rho value, which indicates the patient population for each gene determined. A *p*-value < 0.05 is considered statistically significant.

Our next studies focused on determining the correlation between HDAC4, T-cell inflamed TME gene signatures, and the abundance of TILs, including activated DCs and activated cytotoxic CD8^+^ T-cells. Further analysis of the SKCM dataset using the TISIDB database was performed (Figure 3 and Figure S2). Figure 3A illustrates a modest negative correlation between HDAC4 expression and the abundance of activated DCs and activated cytotoxic CD8^+^ T-cells. The data demonstrate that the elevated expression of the T-cell inflamed signature and the abundance of activated DCs as well as activated cytotoxic CD8^+^ T-cells were positively correlated (Figure 3B). The influence of the type II IFN-γ-related gene signature and T effector signature on the abundance of activated DC and activated cytotoxic CD8^+^ T-cells is shown in Figure S2A,B. These results further emphasize the association between high HDAC4 expression and a less inflamed TME, which may negatively impact immune-cell-mediated tumor killing. We next investigated the influence of HDAC4 on the transcription and DNA methylation of the T-cell inflamed TME gene signatures.

### 3.3. The Effect of HDAC4 Expression on the Transcription of T-Cell Inflamed TME Gene Signatures

**Figure 4:**
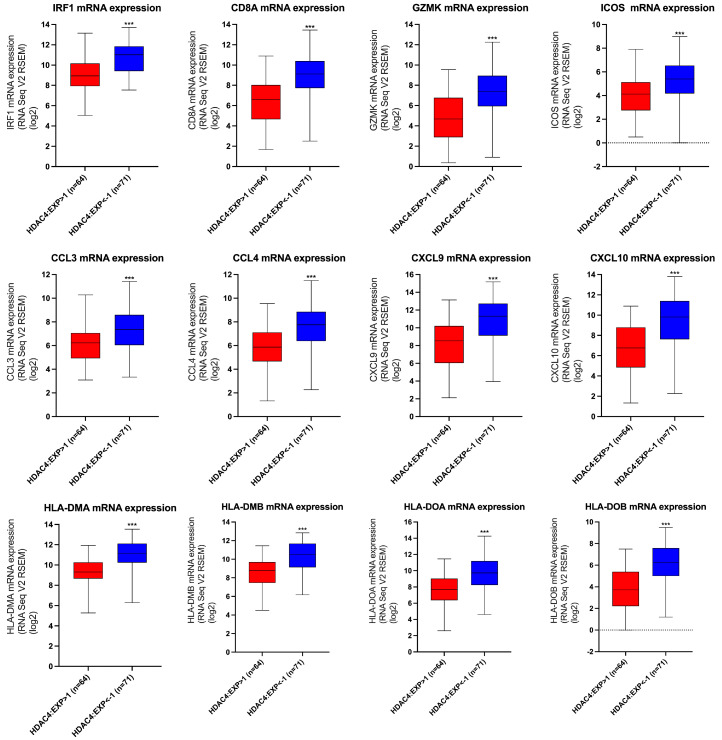
Elevated HDAC4 expression was associated with a low transcription of T-cell inflamed signature. The mRNA expression levels of the T-cell inflamed signature based on HDAC4 expression. Patients are divided into two groups: those with high HDAC4 expression (HDAC4: EXP > 1, n = 64) and those with low HDAC4 expression (HDAC4: EXP < −1, n = 71). Student’s *t*-test was used for the comparison of statistical significance, and *** denotes a *p*-value < 0.001.

Additional analysis was conducted to evaluate the impact of HDAC4 expression on the mRNA expression of T-cell inflamed TME gene signatures. The data were acquired from the SKCM (TCGA, Firehose Legacy) dataset in cBioPortal. Based on their HDAC4 expression, patients were divided into two groups: those with high HDAC4 expression and those with low HDAC4 expression. The mRNA expression levels of the T-cell inflamed signature genes were compared between the two groups. Figure 4 shows that high HDAC4 expression was associated with significantly lower mRNA levels of the T-cell inflamed signature. In addition, Figure S3A,B demonstrates that HDAC4 adversely influenced the transcription of the type II IFN-γ-related gene signature and T effector signature. Overall, our results reinforced the negative regulatory role of HDAC4 on these immune-related genes.

### 3.4. The Effect of HDAC4 Expression on the DNA Methylation of T-Cell Inflamed TME Gene Signatures

**Figure 5:**
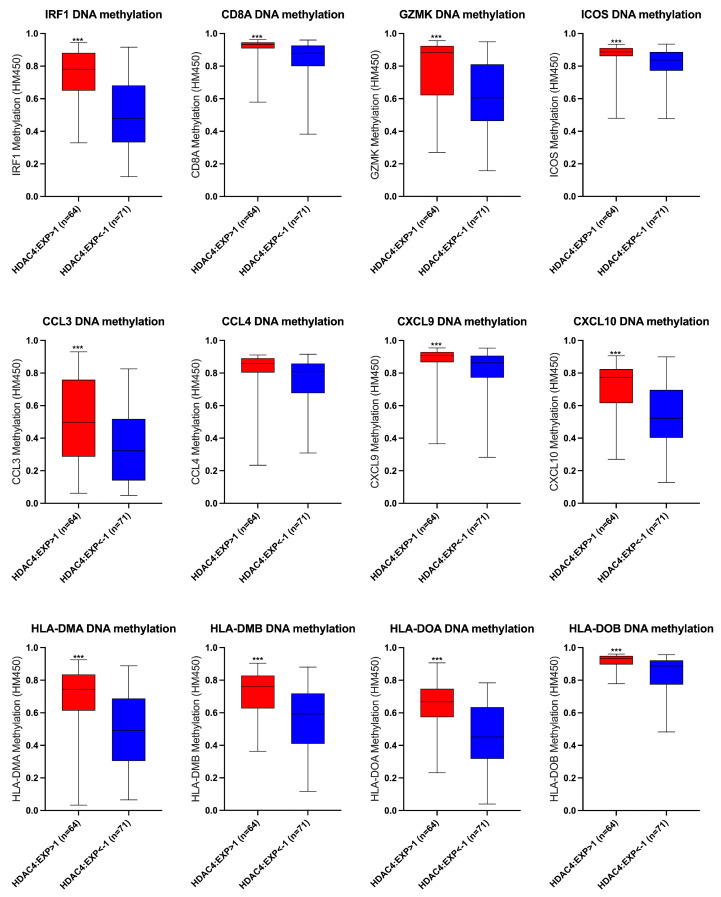
Elevated HDAC4 expression was associated with high DNA methylation levels of the T- cell inflamed signature. The DNA methylation levels of the T-cell inflamed signature based on HDAC4 expression. Patients are divided into two groups: those with high HDAC4 expression (HDAC4: EXP > 1, n = 64) and those with low HDAC4 expression (HDAC4: EXP < −1, n = 71). Student’s *t*-test was used for the comparison of statistical significance, and *** denotes a *p*-value < 0.001.

As DNA methylation is one of the mechanisms causing immune evasion, we next assessed whether elevated HDAC4 expression affected the DNA methylation of the T-cell inflamed TME gene signatures. The patient classification based on HDAC4 levels depicted in Figure 4 was applied for the assessment. The results indicated that that patients with high HDAC4 expression exhibited increased DNA methylation of the T-cell inflamed signature (Figure 5). Additional analysis demonstrated a positive correlation between HDAC4 expression and DNA methylation levels of the type II IFN-γ-related gene signature and T effector signature (Figure S4A,B). These data suggested a potential epigenetic mechanism by which HDAC4 can modulate immune response.

### 3.5. Prognostic Values of HDAC4 and T-Cell Inflamed TME Gene Signatures

**Figure 6:**
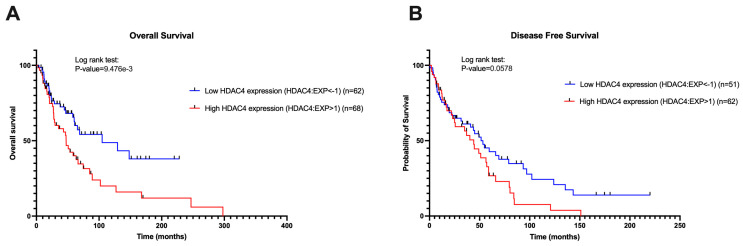
Low HDAC4 expression was associated with a high survival rate. (**A**,**B**) OS and DFS of HDAC4 and T-cell inflamed signature genes in melanoma patients. Patients are categorized based on HDAC4 expression. The log-rank test was used for analysis. The Kaplan–Meier plot was created using GraphPad Prism 10.0, and a *p*-value < 0.05 is considered as statistically significant. The *p*-value of 9.476e-3 denotes significance at <0.001.

We determined the effect of HDAC4 expression on patients’ OS and DFS. Figure 6 presents the survival analysis comparing the two SKCM patient groups depending on HDAC4 expression, as previously illustrated in Figure 4 and Figure 5. The difference in patient numbers between Figure 6 compared to Figure 4 and Figure 5 was due to the lack of survival data for certain patients in the cBioPortal database. The analysis revealed that patients with low HDAC4 expression had better survival outcomes (Figure 6A,B). To enhance the generalizability of our results, Figure S5 illustrated the OS among melanoma patients grouped by high, intermediate, and low HDAC4 expression. This finding highlights the prognostic value of HDAC4, indicating that its lower expression levels predicted more favorable outcomes, likely due to enhanced immune activity within the TME.

Data from the UALCAN database were used to conduct further survival analyses on the SKCM dataset. The patients were classified based on the median of the gene expression. Figure S6 indicates that high HDAC4 expression was associated with poor prognosis in melanoma, while the high expression of T-cell inflamed TME gene signatures correlated with longer survival and better prognosis. This underscored the opposing prognostic implications of HDAC4 and T-cell inflamed TME gene signatures, with HDAC4 serving as a negative prognostic marker and T-cell inflamed TME gene signatures indicating improved patient outcomes.

### 3.6. The Effect of HDAC4 in the ICI-Pretreated Melanoma Dataset

**Figure 7:**
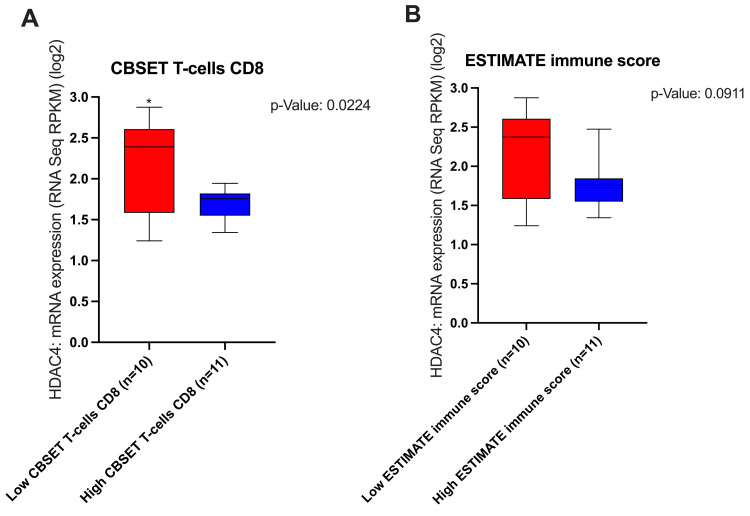
Low HDAC4 expression linked to improved TIL status within the TME in ICI-pretreated melanoma patients. (**A**) The mRNA expression of HDAC4 based on CBSET T-cell CD8. Patients are divided into two CBSET T-cell CD8 groups: low (n = 10) and high (n = 11). (**B**) The mRNA expression of HDAC4 based on the ESTIMATE immune score. Patients are divided into two groups: low (n = 10) and high (n = 11) ESTIMATE immune score. Student’s *t*-test was used for the comparison of statistical significance, and * denotes a *p*-value < 0.05.

The previous detailed analyses elucidated the negative correlation between HDAC4 and T-cell inflamed TME gene signatures and the implications of this relationship on TME, immune cell infiltration, gene transcription, DNA methylation, and patient survival in the SKCM dataset. To further investigate how HDAC4 can influence the TME in a cohort of melanoma patients who were pretreated with ICIs, we utilized the melanoma (MSK, NEJM 2014) dataset from cBioPortal. As shown in Figure 7, the data indicate that HDAC4 adversely affected TILs, as high HDAC4 mRNA expression was associated with low CBSET T-cell CD8 levels and a low ESTIMATE immune score, which is an algorithm utilized for assessing the TIL status.

Our studies in the SKCM dataset and the ICI-pretreated melanoma dataset have provided valuable insights into the possible role of HDAC4 in modulating the TME in melanoma. Therefore, reducing HDAC4 expression may transform the TME into an inflamed phenotype, potentially leading to a positive outcome.

## 4. Discussion

The incidence of cutaneous malignant melanoma has been increasing globally, which poses significant challenges to the health and safety of patients [1,2,3]. As the development of tumor resistance mechanisms and recurrence rates have been attributed to a poor prognosis [3,4,5], the implications of counter-regulatory factors involved in augmenting tumor growth could provide potential strategies to devise alternative approaches to improve clinical outcomes. Given the importance of immune regulation and tumor characteristics in impacting T-cell inflamed TME gene signatures as well as the efficacy of therapeutic agents, including ICIs [36,37,40], deciphering the roles of potential factors involved in interfering with therapy effectiveness could provide novel strategies for melanoma intervention. As HDACs have been shown to be frequently deregulated in melanomas and can modulate the expression of PD-1/PD-L1 and other genes linked to immune evasion [43,44,45,46,47,48], a significant knowledge gap exists between HDAC isoforms and the T-cell inflamed TME signature in terms of predicting the therapeutic responses of ICIs. While a few studies have used in vitro and in vivo melanoma models to determine the effects of pan-HDAC inhibitors [47,62,63], the impact of HDAC4 in melanoma patients has remained elusive. Notably, the combination of belinostat (a pan-HDAC inhibitor) with cisplatin and etoposide exerted hematologic toxicity in a phase I clinical trial involving patients with advanced small cell lung cancer [64]. Thus, the current study was designed to determine the impact of the association between T-cell inflamed TME gene signatures and HDACs, with major emphasis on HDAC4 in influencing the efficacy of ICIs in melanoma patients. This study could offer a more focused approach than employing a pan HDAC inhibitor to overcome the associated adverse effects and improve patient outcomes with the ICI combination.

Analyzing the co-expression profile of the SKCM from the TCGA dataset, we found that HDAC4 expression was negatively correlated with the T-cell inflamed TME gene signatures across the sample population, indicating an inverse relationship between these gene sets in melanoma patients. Further analysis suggested a positive correlation between the increased infiltration of activated DC and cytotoxic CD8^+^ T-cells with T-cell inflamed TME gene signatures, as well as a negative correlation with high HDAC4 expression. The correlation between HDAC4 expression and TILs was modest, as shown in Figure 3A. Our findings indicated that increased HDAC4 expression could result in reduced immune cell infiltration. As the T-cell inflamed TME correlated with pre-existing immune activation, our findings were in agreement with previous reports indicating that T-cell inflamed TME gene signatures were attributed to an increased responsiveness to immunotherapy approaches, including ICIs [38,39,40,41,42,65]. In addition, the T-cell inflamed TME gene signatures we analyzed have also been found to be associated with clinical response and could be used as predictive tools to evaluate patient samples to guide immunotherapy approaches such as PD-1/PD-L1 inhibitors [38,39,40,41,42]. Importantly, while the non-T-cell inflamed TME has been correlated with drug resistance, T-cell inflamed TME gene signatures have also not always provided a clinical benefit from PD-1/PD-L1 blockade [37,65]. This indicates the involvement of distinct mechanisms and/or potential factors that mediated drug resistance and/or treatment failure in T-cell inflamed versus non-T-cell inflamed tumors [66,67,68,69,70].

Notably, HDACs were not only overexpressed or deregulated in multiple cancer models, including melanoma, but they also regulated epigenetic mechanisms and non-histone proteins, including transcription factors and cell signaling pathways [63,71,72]. These changes resulted in altered gene expression and genomic integrity, conferring a poor prognosis, which provided the rationale for developing HDAC inhibitors. Several new molecules have been explored as potential HDAC inhibitors that can target multiple classes of HDACs and were shown to induce a range of antitumor activities, including cell cycle arrest, cell death, tumor differentiation, and increased tumor immunogenicity [73]. For example, vorinostat targets class I, II, and IV HDACs and was approved for the treatment of cutaneous T-cell lymphoma as monotherapy and also in combination with other HDAC inhibitors such as romidepsin and belinostat [74,75,76]. Importantly, isoform-specific HDAC inhibitors such as benzamide derivatives that target class I HDAC have also been developed and evaluated against solid tumors in clinical trials, which showed mixed response rates [73,77].

While several HDAC inhibitors have been tested in combination with ICIs, such as anti-PD-1 and anti-CTLA4 therapy against multiple advanced-stage solid tumors, to the best of our knowledge, the effects of HDAC4 inhibitors with ICIs have not been reported, particularly in melanoma patients. This provided the rationale to extend our studies to explore the impact of HDAC4 expression on the transcription as well as DNA methylation activity of T-cell inflamed TME gene signatures with the goal of correlating their significance with the survival rates of melanoma patients. As DNA methylation has been linked with favoring tumor growth via causing immune evasion, drugs that target this mechanism are being explored as promising strategies for cancer treatment [72,78,79]. Our findings supported that HDAC4 can have a negative regulatory function in immune-related genes, since HDAC4 expression was linked to significantly lower mRNA levels of T-cell inflamed TME gene signatures. In addition, our data revealed that there was a positive correlation between the upregulation of DNA methylation of T-cell inflamed TME gene signatures and high HDAC4 expression, suggesting a possible epigenetic pathway by which HDAC4 can regulate immune response.

Given that HDAC4 expression negatively impacted T-cell inflamed TME gene signatures, we next sought to determine its effect on the prognosis of melanoma patients. The data demonstrated that significantly increased survival rates were associated with low HDAC4 expression. These findings suggested that reduced HDAC4 levels predicted favorable outcomes and high HDAC4 expression correlated with poor prognosis. Overall, these studies highlighted the prognostic value of HDAC4 and indicated that a combination of HDAC4 inhibitor and ICIs would result in a better prognosis for melanoma patients.

To further strengthen our hypothesis, we next evaluated the effect of HDAC4 in the ICI-pretreated cohort of melanoma patients. The data demonstrated that elevated HDAC4 expression could modulate the TME by decreasing the infiltration of CD8^+^ T-cells. This indicates that HDAC4 inhibitors may represent a promising strategy to improve the effect of ICIs in melanoma patients.

Despite these promising insights, several limitations must be addressed. First, our work was primarily based on utilizing several databases and multiple algorithms, similar to other published reports [80,81,82,83]. Thus, rigorous in vitro studies are warranted. However, cell-line-based studies do not fully represent the complexities of human tumors. Second, several in vivo models have been developed; however, often their findings do not translate to human studies [84,85]. Third, identifying the patient population most likely to benefit from such a combination also represents an important area for future research.

## 5. Conclusions

Due to the development of drug resistance and recurrence rates, the treatment of advanced-stage solid tumors, including malignant melanoma, remains challenging. While multiple therapies, including ICIs, are available, the prognosis of melanoma patients remains poor. Thus, the development of new approaches requires the implication of novel strategies to counteract resistance mechanisms leading to tumor immune escape and to guide prospective immunotherapy for patients who do not benefit from single-agent ICIs. Considering the importance of T-cell inflamed TME gene signatures driving TILs, as well as the potential impact of HDAC4 in regulating cancer growth and tumor immunity, the delineation of molecular mechanisms leading to reduced antitumor immune responses is of utmost significance. Given the promising efficacy of pan-HDAC inhibitors in early-phase clinical trials against solid tumors, the implication of HDAC4 inhibitors in combination with ICIs could result in better prognoses. Nevertheless, further analyses using ChIP-seq data, promoter motif enrichment, or histone modification signatures are needed to validate the significance of HDAC4 in melanoma prognosis.

## Data Availability

The authors will provide the raw data that support the conclusions of this article upon request. The datasets analyzed during the study are available in the following repositories (https://www.cbioportal.org/; accessed on 11 November 2024 and on 10 April 2025), (http://timer.cistrome.org/; accessed on 3 December 2024), (http://cis.hku.hk/TISIDB/; accessed on 3 December 2024 and on 9 April 2025), and (https://ualcan.path.uab.edu/index.html; accessed on 11 November 2024).

## Supplementary Materials

The following supporting information can be downloaded at https://www.mdpi.com/article/10.3390/cancers17091518/s1, Figure S1: Hierarchical cluster analysis demonstrated a negative co-expression pattern between HDAC4 and T-cell inflamed TME gene signatures across melanoma patients; Figure S2: High expression of the type II IFN-γ-related gene signature and T effector signature was positively correlated with the abundance of activated DCs and activated cytotoxic CD8^+^ T-cells; Figure S3: High HDAC4 expression resulted in decreased transcription of type II IFN-γ-related gene signature and T effector signature; Figure S4: High HDAC4 expression resulted in increased DNA methylation of type II IFN-γ-related gene signature and T effector signature; Figure S5: High HDAC4 expression correlated with poor prognosis among melanoma patients grouped by low, intermediate, and high HDAC4 expression; Figure S6: Low HDAC4 expression and high T-cell inflamed TME gene signature expression were associated with improved melanoma patient prognosis.

## Abbreviations

The following abbreviations are used in this manuscript:

CCL	C-C Motif Chemokine Ligand
CD	Cluster of Differentiation
CMKLR	Chemerin Chemokine-like Receptor 1
CTLs	Cytotoxic T-cells
CTLA4	Cytotoxic T-Lymphocyte-Associated Protein 4
CXCL	C-X-C Motif Chemokine Ligand
CXCR	C-X-C Motif Chemokine Receptor
DCs	Dendritic Cells
DFS	Disease-Free Survival
GZM	Granzyme
HDACs	Histone Deacetylases
HLA	Major Histocompatibility Complex, class II
IC	Immune Checkpoint
ICIs	Immune Checkpoint Inhibitors
ICOS	Inducible T-Cell Co-stimulator
IDO1	Indoleamine 2,3-dioxygenase
IFN	Interferon
IRF1	IFN Regulatory Factor 1
LAG3	Lymphocyte Activation Gene 3
mDCs	Myeloid Dendritic Cells
MDSCs	Myeloid-Derived Suppressor Cells
NK	Natural Killer Cells
NKG7	Natural Killer Cell Granule Protein 7
OS	Overall Survival
pDC	Plasmacytoid Dendritic Cells
PD-L1	Programmed Cell Death Ligand 1
PD-1	Programmed Cell Death Protein 1
PRF1	Perforin 1
PSMB10	Proteasome 20S Subunit, Beta type, 10
SKCM	Skin Cutaneous Melanoma
STAT1	Signal Transducers and Activators of Transcription 1
TCGA	The Cancer Genome Atlas
TH	T-helper Cells
Treg	Regulatory T-cells
TIGIT	T-Cell Immunoreceptor with Ig and ITIM Domains
TILs	Tumor-Infiltrating Lymphocytes
TIM-3	T-cell Immunoglobulin and Mucin-domain containing-3
TLTs	Tertiary Lymphoid Tissues
TMB	Tumor Mutational Burden
TME	Tumor Microenvironment
