# Supplementary material for: Elevated HDAC4 Expression Is Associated with Reduced T-Cell Inflamed Tumor Microenvironment Gene Signatures and Immune Checkpoint Inhibitor Effectiveness in Melanoma
Source: Cancers (Basel). 2025 Apr 30;17(9):1518. doi: 10.3390/cancers17091518 (PMC12070970; doi:10.3390/cancers17091518)
Supplement: Supplementary file 1 [file cancers-17-01518-s001.zip › Figure S1.pdf]

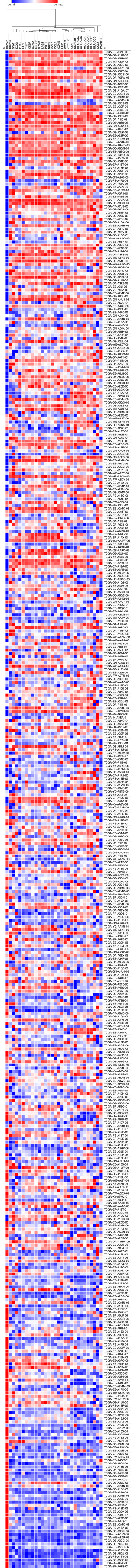

Figure S1: Hierarchical cluster analysis demonstrated a negative co-expression pattern between HDAC4, and T-cell inflamed TME gene signatures across melanoma patients.
