# Supplementary material for: Elevated HDAC4 Expression Is Associated with Reduced T-Cell Inflamed Tumor Microenvironment Gene Signatures and Immune Checkpoint Inhibitor Effectiveness in Melanoma
Source: Cancers (Basel). 2025 Apr 30;17(9):1518. doi: 10.3390/cancers17091518 (PMC12070970; doi:10.3390/cancers17091518)
Supplement: Supplementary file 1 [file cancers-17-01518-s001.zip › Figure S2.pdf]

A) Type II IFN-γ- related gene signature

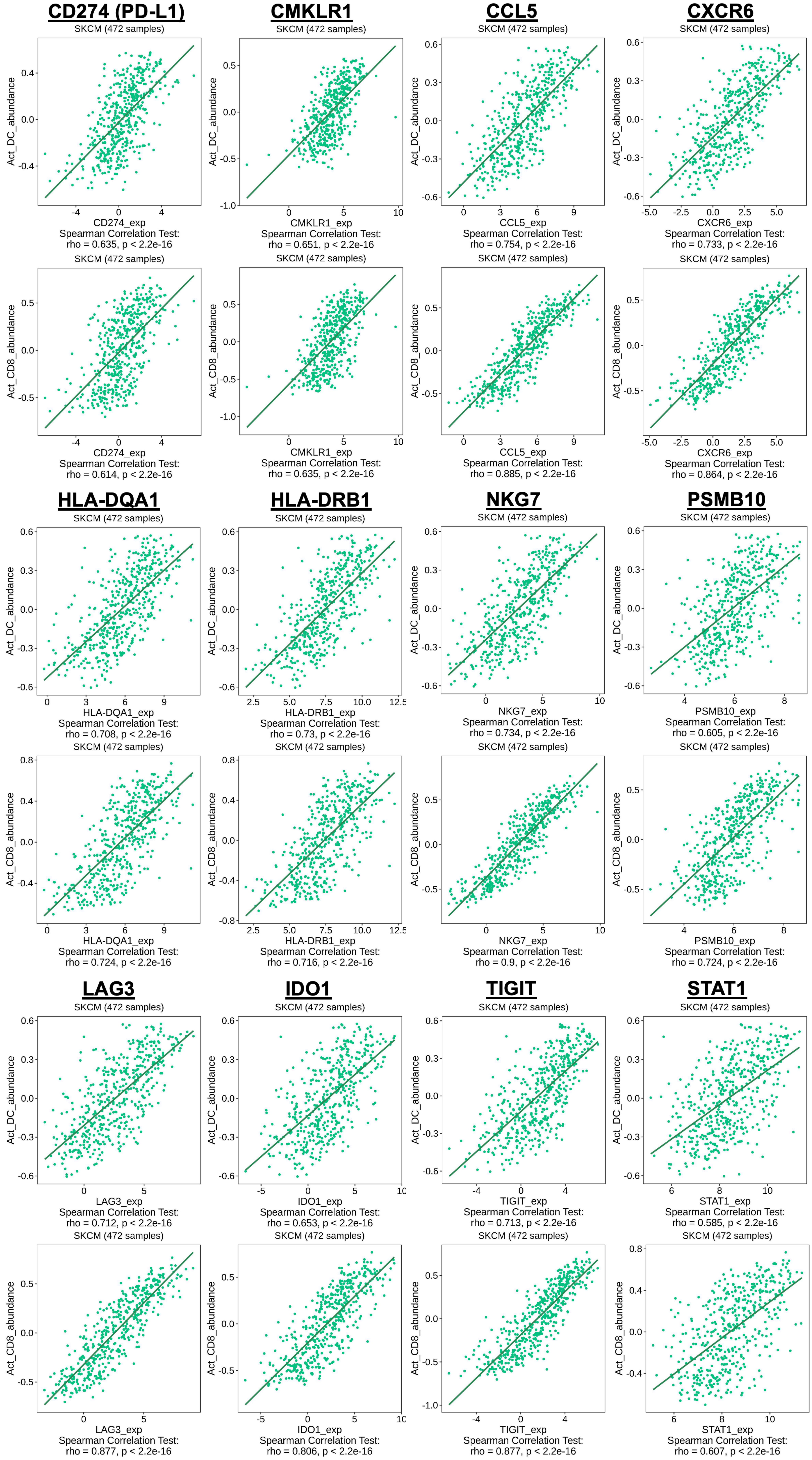

B) T effector signature

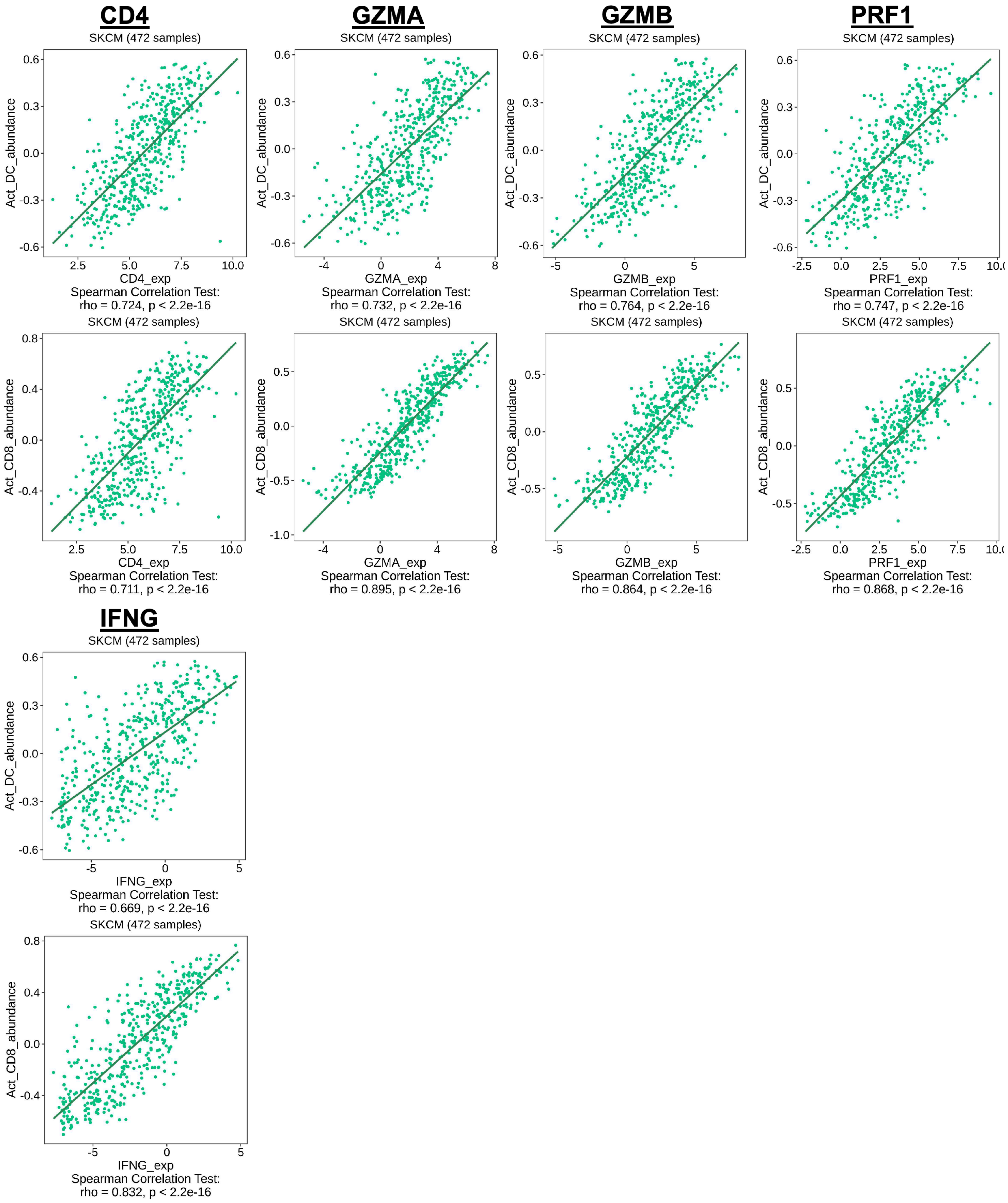

Figure S2: High expression of type II IFN-γ-related gene signature and T effector signature was positively correlated with the abundance of activated DCs and activated cytotoxic CD8+ T-cells.
