# Supplementary material for: Elevated HDAC4 Expression Is Associated with Reduced T-Cell Inflamed Tumor Microenvironment Gene Signatures and Immune Checkpoint Inhibitor Effectiveness in Melanoma
Source: Cancers (Basel). 2025 Apr 30;17(9):1518. doi: 10.3390/cancers17091518 (PMC12070970; doi:10.3390/cancers17091518)
Supplement: Supplementary file 1 [file cancers-17-01518-s001.zip › Figure S4.pdf]

A) Type II IFN-γ-related gene signature

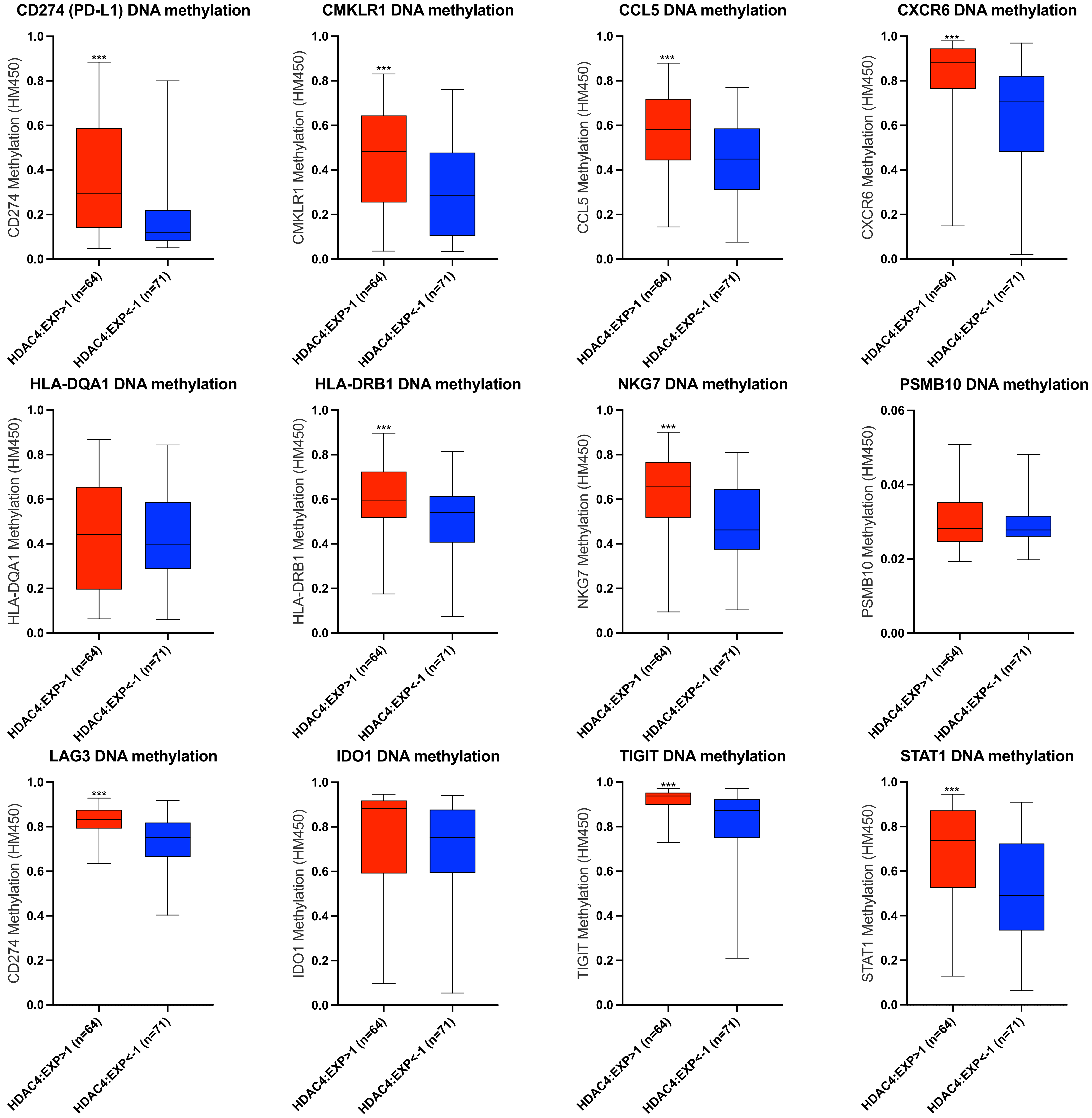

B) T effector signature

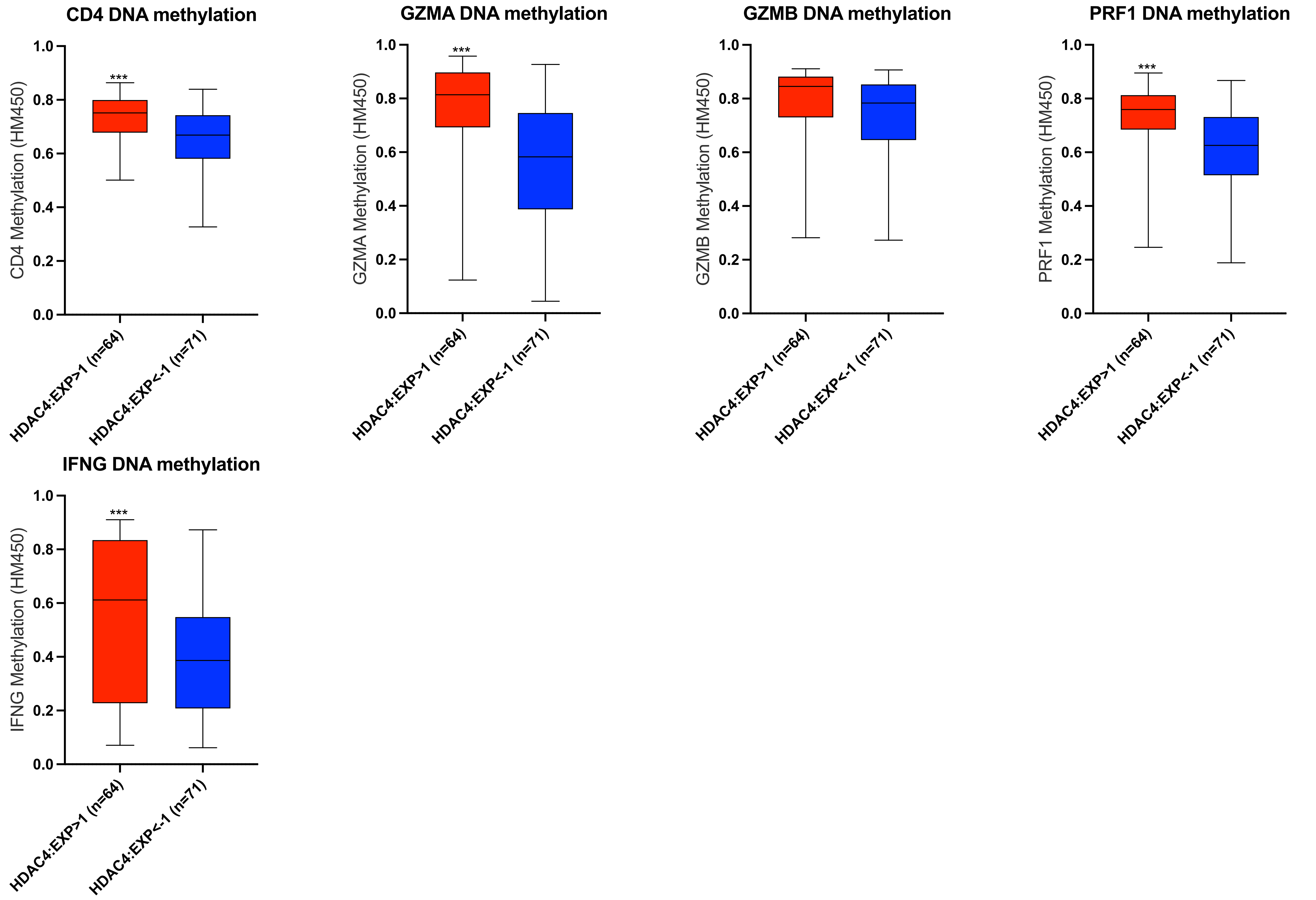

Figure S4: High HDAC4 expression resulted in increased DNA methylation of type II IFN-γ-related gene signature and T effector signature.
