# Supplementary material for: Elevated HDAC4 Expression Is Associated with Reduced T-Cell Inflamed Tumor Microenvironment Gene Signatures and Immune Checkpoint Inhibitor Effectiveness in Melanoma
Source: Cancers (Basel). 2025 Apr 30;17(9):1518. doi: 10.3390/cancers17091518 (PMC12070970; doi:10.3390/cancers17091518)
Supplement: Supplementary file 1 [file cancers-17-01518-s001.zip › Figure S6.pdf]

A) HDAC4

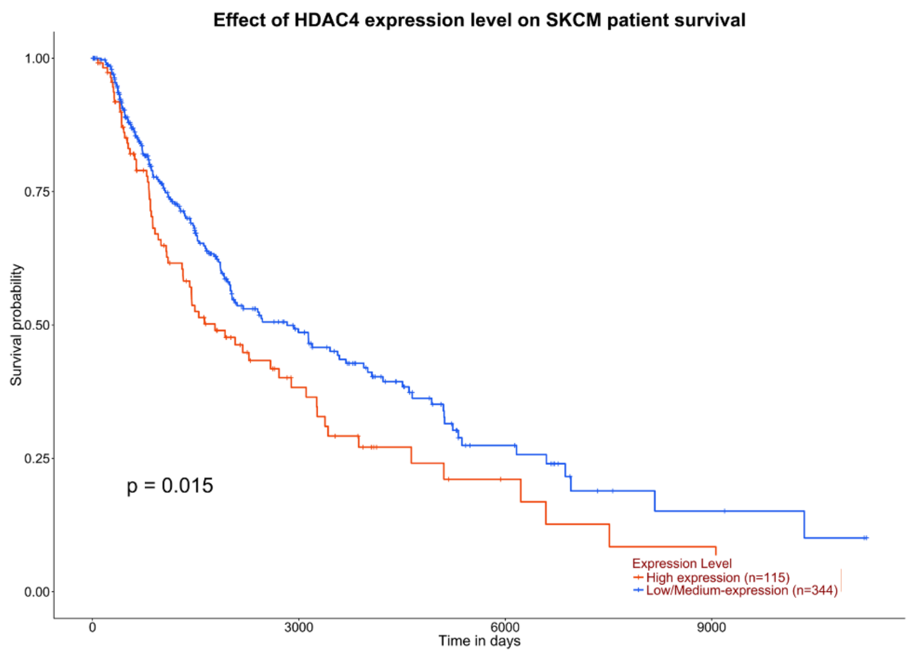

B) T-cell inflamed Signature

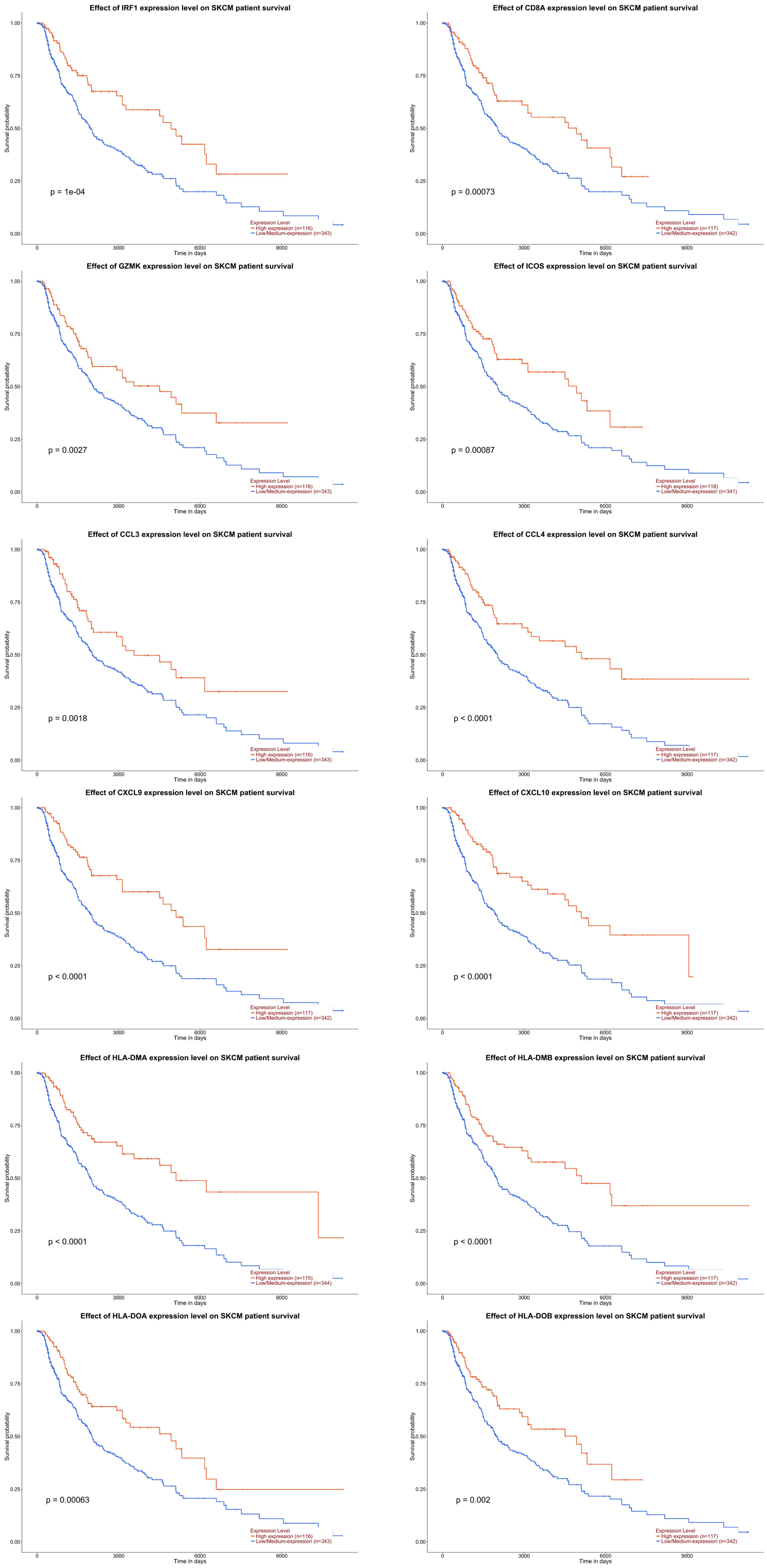

C) Type II IFN-γ- related gene signature

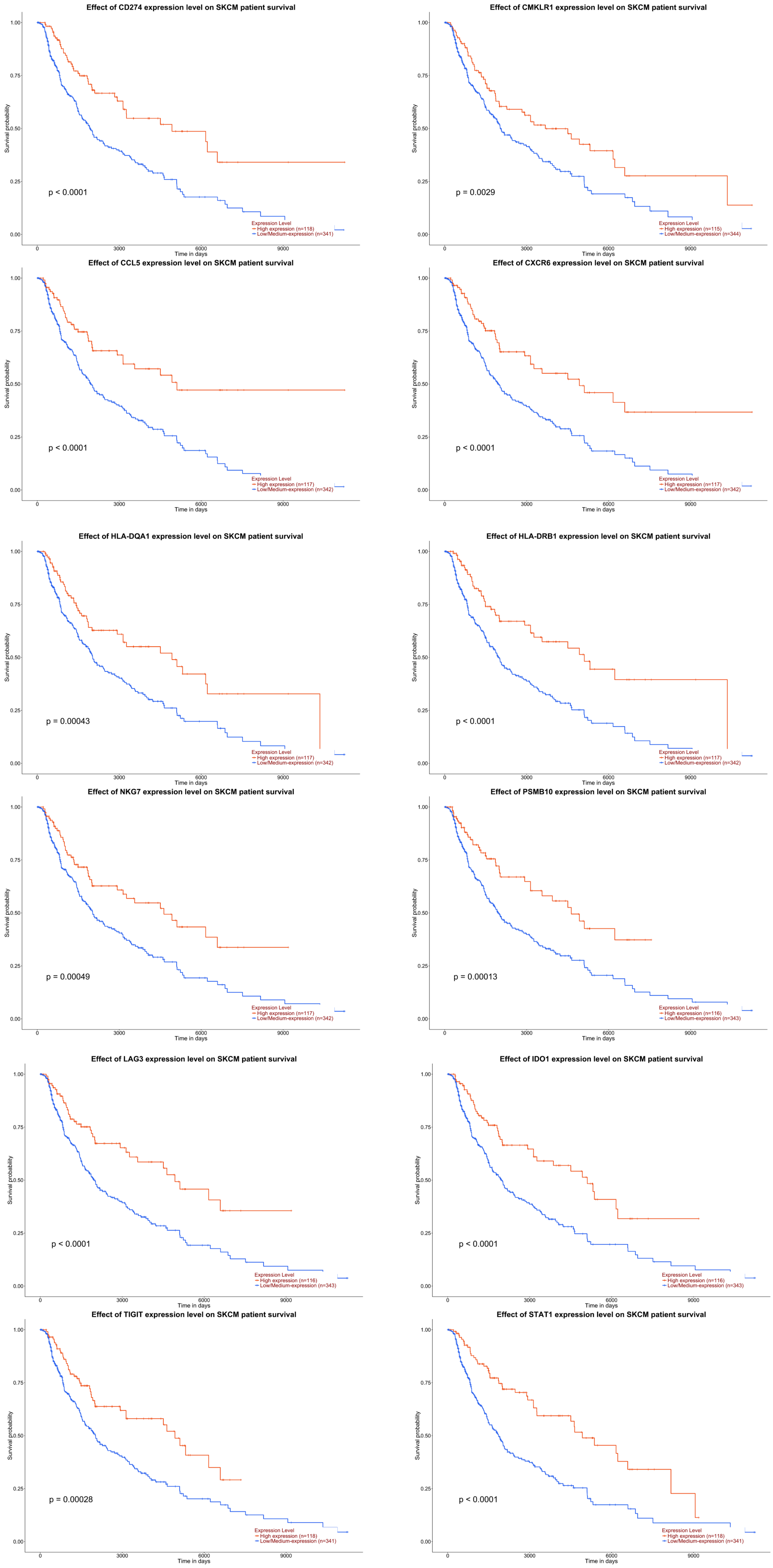

D) T effector signature

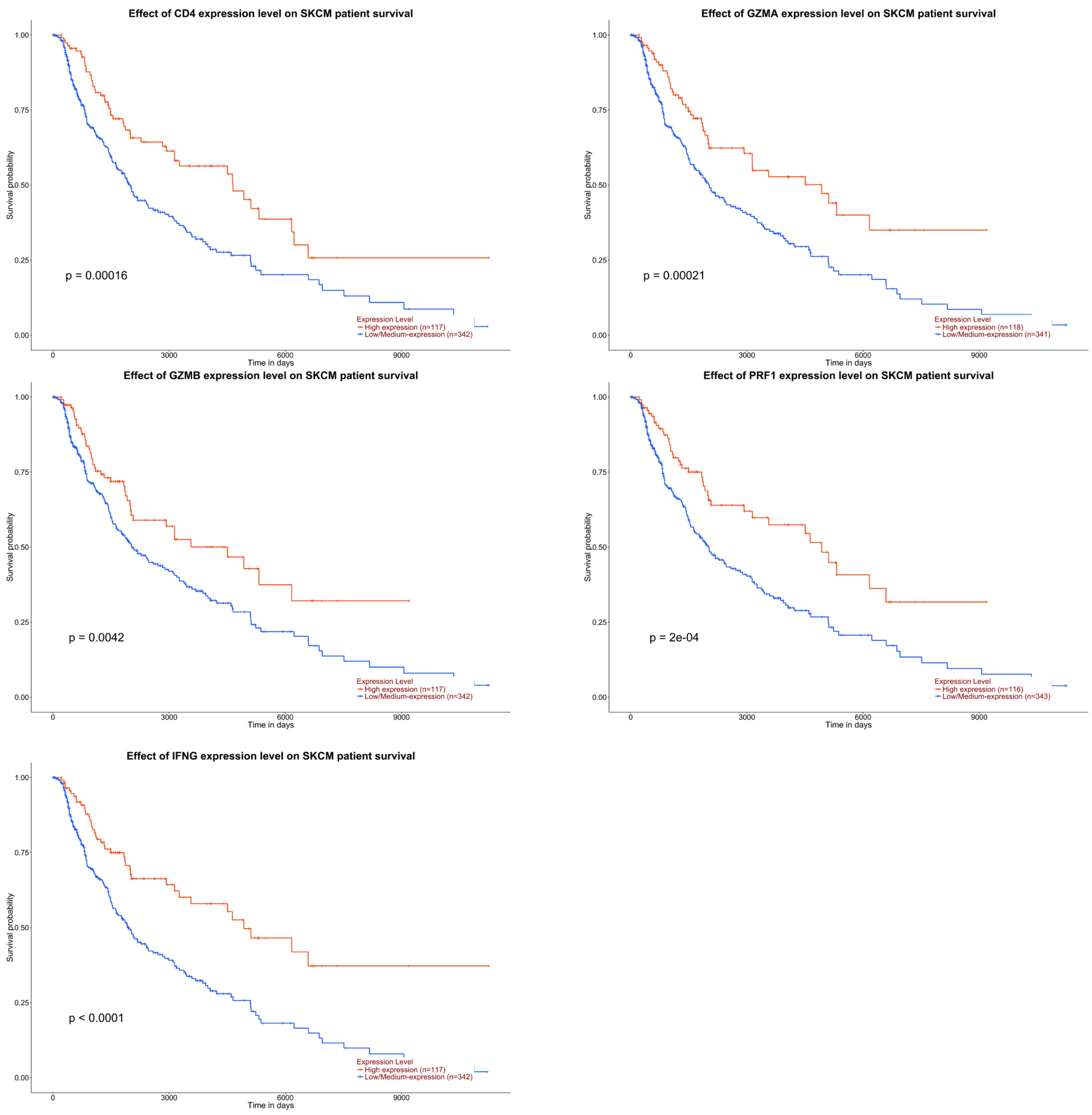

Figure S6: Low HDAC4 expression and high T-cell inflamed TME gene signatures expression were associated with improved melanoma patients' prognosis.
